# Supplementary material for: Lung Cancer as a Leading Cause among Paraneoplastic Non-Bacterial Thrombotic Endocarditis: A Meta-Analysis of Individual Patients’ Data
Source: Cancers (Basel). 2023 Mar 20;15(6):1848. doi: 10.3390/cancers15061848 (PMC10047261; doi:10.3390/cancers15061848)

# Supplementary Materials

## Supplementary Table S1: Searched keywords table

| # | Search |
|---|---|
| **1** | “endocarditis, non infective” [MeSH Terms] |
| **2** | “endocarditis” [All Fields] |
| **3** | “non infective” [All Fields] |
| **4** | 2 AND 3 |
| **5** | “non-infective endocarditis” [All Fields] |
| **6** | “non” [All Fields] |
| **7** | “bacterial” [All Fields] |
| **8** | “thrombotic” [All Fields] |
| **9** | 6 AND 7 AND 8 AND 2 |
| **10** | “marantic endocarditis” [All Fields] |
| **11** | “marantic” [All Fields] |
| **12** | 11 AND 2 |
| **13** | “trousseau” [All Fields] OR “trousseau s” [All Fields]) |
| **14** | “syndrome” [All Fields] OR “syndromal” [All Fields] OR “syndromally” [All Fields] OR “syndrome” [MeSH Terms] OR “syndrome” [All Fields] OR “syndromes” [All Fields] OR “syndrome s” [All Fields] OR “syndromic” [All Fields] OR “syndroms” [All Fields] |
| **15** | 13 AND 14 |
| **16** | "cancer s"[All Fields] OR "cancerated"[All Fields] OR "canceration"[All Fields] OR "cancerization"[All Fields] OR "cancerized"[All Fields] OR "cancerous"[All Fields] OR "neoplasms"[MeSH Terms] OR "neoplasms"[All Fields] OR "cancer"[All Fields] OR "cancers"[All Fields] |
| **17** | 1 OR 4 OR 9 OR 12 OR 15 |
| **18** | 16 AND 17 |

**Supplementary Table S2: Quality assessment of the included studies ¶**

| | Country | Q1 ¶ | Q2 | Q3 | Q4 | Q5 | Q6 | Q7 | Q8 |
|---|---|---|---|---|---|---|---|---|---|
| **Ahmed 2018(22)** | USA | Yes | Yes | Yes | Yes | Yes | Yes | Yes | Yes |
| **Alaiti 2015(23)** | USA | Yes | Yes | No | Yes | Yes | Unclear | Yes | Yes |
| **Albright 2016(24)** | USA | Yes | No | Yes | Yes | Yes | Yes | Yes | Yes |
| **Ali 2012(25)** | Australia | Yes | Yes | Yes | Yes | Yes | Unclear | Yes | Yes |
| **Ali 2015(26)** | UK | Yes | Yes | Yes | Yes | Yes | Yes | Yes | Yes |
| **Arvold 2011(27)** | USA | Yes | Yes | Yes | Yes | Yes | Yes | Yes | Yes |
| **Ashenhurst 1962(28)** | Canada | Yes | Yes | Yes | Yes | Yes | Yes | Yes | Yes |
| **Bhardwaj 2016(29)** | USA | Yes | Yes | Yes | Yes | Yes | Yes | Yes | Yes |
| **Binet 2021(30)** | Belgium | Yes | Yes | Yes | Yes | Yes | Yes | Yes | Yes |
| **Borowski 2005(31)** | Germany | Yes | Yes | Yes | Yes | Yes | Yes | Yes | Yes |
| **Chen 2004(32)** | USA | Yes | Yes | Yes | Yes | Yes | Yes | Yes | Yes |
| **Cheung 2020(33)** | USA | Yes | Yes | Yes | Yes | Yes | Yes | Yes | Yes |
| **Chisholm 1982(34)** | USA | Yes | Yes | Yes | Yes | Yes | Yes | Yes | Yes |
| **Clough 2010(35)** | UK | Yes | Yes | Yes | Yes | Yes | Yes | Yes | Yes |
| **Detremerie 2017 (36)** | Belgium | Yes | Yes | Yes | Yes | Yes | Yes | Yes | Yes |
| **Dewey 2014(37)** | UK | Yes | Yes | Yes | Yes | Yes | Unclear | Yes | No |
| **Douin 2020(38)** | Italy | Yes | Yes | Yes | Yes | Yes | No | Yes | Yes |
| **Elboudwarej 2015(39)** | USA | Yes | Yes | Yes | Yes | Yes | Yes | Unclear | No |
| **Fanale 2002(40)** | Taiwan | Yes | Yes | Yes | Yes | Yes | Yes | Yes | Yes |
| **Farooqui 2021(41)** | USA | Yes | Yes | Yes | Yes | Yes | Yes | Yes | Yes |
| **Ferreira 2017(42)** | Portugal | Yes | Yes | Yes | Yes | Yes | Yes | Yes | Yes |
| **Fournier 2019(43)** | USA | Yes | Yes | Yes | Yes | Yes | Yes | Unclear | No |
| **Frazer 2005(44)** | UK | Yes | Yes | Yes | Yes | Yes | No | No | Yes |
| **Fujimoto 2018(45)** | Japan | Yes | Yes | Yes | Yes | Yes | Yes | Yes | No |
| **Garcia 1983(46)** | USA | Yes | Yes | Yes | Yes | Yes | Yes | Yes | Yes |
| **Glass 1993(47)** | USA | Yes | Yes | Yes | Yes | Yes | Yes | Yes | Yes |
| **Gray 2021(48)** | USA | Yes | Yes | Yes | Yes | Yes | Yes | Yes | Yes |
| **Green 1990(49)** | Scotland | Yes | Yes | Yes | Yes | No | Yes | No | Yes |
| **Gundersen 2016 (50)** | UK | Yes | Yes | Yes | Yes | Yes | Yes | Yes | Yes |
| **Harris 2021(51)** | New Zealand | Yes | Yes | Yes | Yes | Yes | Yes | Yes | Yes |
| **Hofstra 2009(52)** | Netherlands | Yes | Yes | Yes | Yes | Yes | Yes | Unclear | No |
| **Holt 2021(53)** | Australia | Yes | Yes | Yes | Yes | Yes | Yes | Yes | Yes |
| **Ilyas 2021(54)** | Rhode Island | Yes | Yes | Yes | Yes | Yes | Yes | Yes | Yes |
| **Iranpour 2013(55)** | Iran | Yes | Yes | Yes | Yes | Yes | Yes | Yes | Yes |
| **Ito 2013(56)** | Japan | Yes | Yes | Yes | Yes | Yes | Yes | Yes | Yes |
| **Jameson 2009(57)** | USA | Yes | Yes | Yes | Yes | Yes | Yes | Yes | Yes |

| | Country | Q1 ¶ | Q2 | Q3 | Q4 | Q5 | Q6 | Q7 | Q8 |
|---|---|---|---|---|---|---|---|---|---|
| **Joshi 2009(58)** | USA | Yes | Yes | Yes | Yes | Yes | Yes | Yes | Yes |
| **Julson 2018 (59)** | USA | No | No | No | No | No | Yes | Yes | Yes |
| **Kalangos 1997(60)** | Switzerland | Yes | Yes | Yes | Yes | Yes | Yes | Yes | Yes |
| **Kaneyuki 2017(61)** | Japan | Yes | Yes | Yes | Yes | Yes | Yes | Yes | Yes |
| **Kanthasamy 2016(62)** | UK | Yes | Yes | Yes | Yes | Yes | Yes | Yes | Yes |
| **Kaufmann 2020(63)** | Austria | Yes | Yes | Yes | Yes | Yes | Yes | Yes | Yes |
| **Kearsley 1982(64)** | Australia | Yes | Yes | Yes | Yes | Yes | Yes | Yes | Yes |
| **Khan 2008 (65)** | UK | Yes | Yes | Yes | Yes | Yes | Yes | Yes | Yes |
| **Khan 2019(66)** | Pakistan | Yes | Yes | Yes | Yes | Yes | Yes | Yes | Yes |
| **Kijpaisalratanaa 2020(67)** | Thailand | Yes | Yes | Yes | Yes | Yes | Yes | Yes | Yes |
| **Kimyai-Asadi 2001(68)** | USA | Yes | Yes | Yes | Yes | Yes | Yes | Yes | Yes |
| **Kooiker 1976(69)** | USA | Yes | Yes | Yes | Yes | Yes | Yes | Yes | Yes |
| **Kuipers 2021(70)** | Netherlands | Yes | Yes | Yes | Yes | Yes | Yes | Yes | Yes |
| **Kurdi 2004 (71)** | Canada | Yes | Yes | Yes | Yes | Yes | Yes | Yes | Yes |
| **Kwon 2016(72)** | Korea | Yes | Yes | Yes | Yes | Yes | Yes | Yes | No |
| **Lal 2003(73)** | USA | Yes | Yes | Yes | Yes | Yes | Yes | Yes | Yes |
| **Lee 2012 (74)** | Australia | Yes | Yes | Yes | Yes | Yes | Yes | Yes | Yes |
| **Lee 2014(75)** | Korea | Yes | Yes | Yes | Yes | Yes | Yes | Yes | Yes |
| **Makhdumi 2021(76)** | USA | Yes | Yes | Yes | Yes | Yes | No | Unclear | Yes |
| **Mantovani 2016(77)** | Italy | Yes | Yes | Yes | Yes | Yes | Yes | Yes | Yes |
| **Marglani 2009(78)** | Canada | Yes | Yes | Yes | Yes | Yes | Yes | Yes | Yes |
| **Markides 2000(79)** | UK | Yes | Yes | Yes | Yes | Yes | Yes | Yes | Yes |
| **Martín-Martorell 2007(80)** | Spain | Yes | Yes | Yes | Yes | Yes | Yes | Yes | Yes |
| **Mitma 2016(81)** | USA | Yes | Yes | Yes | Yes | Yes | Yes | Yes | Yes |
| **Morimoto 2016 (82)** | Japan | Yes | Yes | Yes | Yes | Yes | Yes | Yes | No |
| **Moţăţăianu 2018(83)** | Romania | Yes | Yes | Yes | Yes | Yes | Yes | Yes | Yes |
| **Nadkarni 2020 (84)** | USA | Yes | Yes | Yes | Yes | Yes | Yes | Yes | No |
| **Nakashima 2012(85)** | Japan | Yes | Yes | Yes | Yes | Yes | Yes | Yes | Yes |
| **Neilan 2018(86)** | USA | Yes | Yes | Yes | Yes | Yes | Yes | Yes | Yes |
| **Al Nidawi 2021(87)** | Kingdom of Bahrain | Yes | Yes | Yes | Yes | Yes | Yes | Yes | Yes |
| **Numnum 2006 (88)** | USA | Yes | Yes | Yes | Yes | Yes | Yes | Yes | Yes |
| **O'boyle 1981(89)** | Ireland | Yes | Yes | Yes | Yes | Yes | Yes | Yes | Yes |
| **Ojeda 1985(90)** | Australia | Yes | Yes | Yes | Yes | Yes | Yes | Yes | Yes |
| **Okuchi 1997(91)** | Japan | Yes | Yes | Yes | Yes | Yes | Yes | Yes | Yes |
| **Olney 1979(92)** | USA | Yes | Yes | Yes | Yes | Yes | Yes | Yes | Yes |
| **Orfanelli 2016(93)** | USA | Yes | Yes | Yes | Yes | Yes | Yes | Yes | Yes |
| **Oueida 2011(94)** | USA | Yes | Yes | Yes | Yes | Yes | Yes | Yes | Yes |

| | Country | Q1 ¶ | Q2 | Q3 | Q4 | Q5 | Q6 | Q7 | Q8 |
|---|---|---|---|---|---|---|---|---|---|
| **Panicucci 2020 (95)** | France | Yes | Yes | Yes | Yes | Yes | Yes | Yes | Yes |
| **Patel 2020(19)** | USA | Yes | Yes | Yes | Yes | Yes | Yes | Yes | Yes |
| **Perrone 2020(96)** | Italy | Yes | Yes | Yes | Yes | Yes | Yes | Yes | Yes |
| **Piovanelli 2013(97)** | Italy | Yes | Yes | Yes | Yes | Yes | Yes | Yes | Yes |
| **Polo 2021(98)** | France | Yes | Yes | Yes | Yes | Yes | Yes | Yes | Yes |
| **Ramirez-Escudero Ugalde 2020(99)** | Spain | Yes | Yes | Yes | Yes | Yes | No | Unclear | Yes |
| **Randhawa 2020(100)** | USA | Yes | Yes | Yes | Yes | Yes | No | Yes | Yes |
| **Royter 2006(101)** | USA | Yes | Yes | Yes | Yes | Yes | Yes | Yes | Yes |
| **Sakima 2011(102)** | Japan | Yes | Yes | Yes | Yes | Yes | No | Unclear | Yes |
| **Sanchez-Quirós 2020(103)** | Spain | Yes | Yes | Yes | Yes | Yes | Yes | Yes | Yes |
| **Sánchez-Enrique 2014(104)** | Spain | Yes | Yes | Yes | Yes | Yes | Yes | Yes | Yes |
| **Savarapu 2021(105)** | USA | Yes | Yes | Yes | Yes | Yes | Yes | Yes | Yes |
| **Sawai 2018(106)** | Japan | Yes | Yes | Yes | Yes | Yes | Yes | Yes | Yes |
| **Scalia 2012(107)** | Australia | Yes | Yes | Yes | Yes | Yes | Yes | Yes | Yes |
| **Sekulic 2020(108)** | USA | Yes | Yes | Yes | Yes | Yes | Yes | Yes | Yes |
| **Shatila 2014(109)** | Lebanon | Yes | Yes | Yes | Yes | Yes | Unclear | Yes | Yes |
| **Shibata 2018(110)** | Japan | Yes | Yes | Yes | Yes | Yes | Yes | Yes | Yes |
| **Shoji 2019(111)** | USA | Yes | Yes | Yes | Yes | Yes | Yes | Yes | Yes |
| **Shuaib, 1991(112)** | Canada | Yes | Yes | Yes | Yes | Yes | Yes | Yes | Yes |
| **Sia 2016(113)** | Singapore | Yes | Yes | Yes | Yes | Yes | Yes | Yes | Yes |
| **Singh 2007(114)** | USA | Yes | Yes | Yes | Yes | Yes | Yes | Yes | Yes |
| **Smeglin 2008(115)** | USA | Yes | Yes | Yes | Yes | Yes | Yes | Yes | Yes |
| **Soga 2018(116)** | Japan | Yes | Yes | Yes | Yes | Yes | Yes | Yes | Yes |
| **Spurgeon 2021(117)** | UK | Yes | Yes | Yes | Yes | Yes | Yes | Yes | Yes |
| **Starobinska 2018(118)** | USA | Yes | Yes | Yes | Yes | Yes | Yes | Yes | Yes |
| **Studdy 1976(119)** | UK | Yes | Yes | Yes | Yes | Yes | Yes | Yes | Yes |
| **Sugawara 2021(120)** | Japan | Yes | Yes | Yes | Yes | Yes | Yes | Yes | Yes |
| **Suzuki 2002(121)** | Japan | Yes | Yes | Yes | Yes | Yes | Yes | Yes | Yes |
| **Tai 2016(122)** | Malaysia | Yes | Yes | Yes | Yes | Yes | Yes | Yes | Yes |
| **Takeshita 2018(123)** | Japan | Yes | Yes | Yes | Yes | Yes | Yes | Yes | Yes |
| **Tamura 2021(124)** | Japan | Yes | Yes | Yes | Yes | Yes | Yes | Yes | Yes |
| **Tiong 2012(125)** | New Zealand | Yes | Yes | Yes | Yes | Yes | Yes | Yes | Yes |
| **Tsai 2015 (126)** | Taiwan | Yes | Yes | Yes | Yes | Yes | Yes | Yes | Yes |
| **Umeojiako 2019(127)** | UK | Yes | Yes | Yes | Yes | Yes | Yes | Yes | Yes |
| **Vaideeswar 1993(128)** | India | Yes | Yes | No | No | No | Yes | Yes | Yes |
| **Van Herck 2021(129)** | Belgium | Yes | Yes | Yes | Yes | Yes | Yes | Yes | Yes |
| **Vlachostergios 2010(130)** | Greece | Yes | Yes | Yes | Yes | Yes | Yes | Yes | Yes |

| | Country | Q1 ¶ | Q2 | Q3 | Q4 | Q5 | Q6 | Q7 | Q8 |
|---|---|---|---|---|---|---|---|---|---|
| **Wang 2020 (131)** | USA | Yes | Yes | Yes | Yes | Yes | Yes | Yes | Yes |
| **Washburn 2016(132)** | USA | Yes | Yes | Yes | Yes | Yes | Yes | Yes | Yes |
| **Wigger 2016(133)** | Switzerland | Yes | Yes | Yes | Yes | Yes | Yes | Yes | Yes |
| **Wild 2021 (134)** | Germany | Yes | Yes | Yes | Yes | Yes | Yes | Yes | Yes |
| **Wong 2013(135)** | Australia | Yes | Yes | Yes | Yes | Yes | Yes | Yes | Yes |
| **Woo 2014(136)** | China | Yes | Yes | Yes | Yes | Yes | Yes | Yes | Yes |
| **Yagi 2014(137)** | Japan | Yes | Yes | Yes | Yes | Yes | Yes | Yes | Yes |
| **Yamane 2014(138)** | Japan | Yes | Yes | Yes | Yes | Yes | Yes | Yes | Yes |
| **Yasutake 2016(139)** | Japan | Yes | Yes | Yes | Yes | Yes | Yes | Yes | Yes |
| **Yoshii 2014(140)** | Japan | Yes | Yes | Yes | Yes | Yes | Yes | Yes | Yes |
| **Zhou 2021(141)** | Singapore | Yes | Yes | Yes | Yes | Yes | Yes | Yes | Yes |

¶ Q1: patient's demographic characteristics, Q2 patient's history and its timeline, Q3 current clinical condition of the patient on presentation, Q4 diagnostic tests or assessment methods and results, Q5 intervention(s) or treatment procedure(s), Q6 post-intervention clinical condition, Q7 adverse events (harms) or unanticipated events, and Q8 takeaway lessons, with each question was answered by either (yes, no, unclear, or not applicable)

**Supplementary Table S3: Different criteria among A) females vs males, and B) marantic first presentation vs known cancer**

| | | A) females vs males | | | B) marantic first presentation vs known cancer | | |
|---|---|---|---|---|---|---|---|
| | **Level** | **Female** | **Male** | **p** | **Known cancer** | **Marantic first presentation** | **p** |
| **n** | | 81 | 63 | | 42 | 83 | |
| **Antiphospholipid antibodies (%)** | No | 79(97.5) | 62(98.4) | 1 | 42(100.0) | 80(96.4) | 0.53 |
| | Yes | 2(2.5) | 1(1.6) | | 0(0.0) | 3(3.6) | |
| **Primary Cancer Organ (%)** | Gynecological | 23(28.4) | 0(0.0) | <0.001 | 11(26.2) | 12(14.5) | 0.312 |
| | Lung | 18(22.2) | 23(36.5) | | 8(19.0) | 23(27.7) | |
| | Other GIT Cancers | 11(13.6) | 8(12.7) | | 6(14.3) | 11(13.3) | |
| | Others | 12(14.8) | 21(33.3) | | 12(28.6) | 19(22.9) | |
| | Pancreas | 17(21.0) | 11(17.5) | | 5(11.9) | 18(21.7) | |
| **Lung vs Other Cancers (%)** | Others | 63(77.8) | 40(63.5) | 0.089 | 34(81.0) | 60(72.3) | 0.401 |
| | Lung cancer | 18(22.2) | 23(36.5) | | 8(19.0) | 23(27.7) | |
| **Pathology (%)** | Adenocarcinoma | 57(70.4) | 36(57.1) | 0.118 | 19(45.2) | 60(72.3) | 0.015 |
| | Hematopoietic | 1(1.2) | 5(7.9) | | 4(9.5) | 1(1.2) | |
| | Others | 21(25.9) | 17(27) | | 16(38.1) | 20(24.1) | |
| | Sarcoma | 0(0.0) | 1(1.6) | | 1(2.4) | 0(0.0) | |
| | SCC | 2(2.5) | 4(6.3) | | 2(4.8) | 2(2.4) | |
| **Adenocarcinoma vs other pathologies (%)** | Other pathologies | 24(29.6) | 27(42.9) | 0.141 | 23(54.8) | 23(27.7) | 0.006 |
| | Adenocarcinoma | 57(70.4) | 36 (57.1) | | 19(45.2) | 60(72.3) | |
| **Organ of metastasis (n=97) (%)** | Bone | 0 (0.0) | 5 (10.6) | 0.246 | 0 (0.0) | 4 (7.4) | 0.085 |
| | Brain | 1 (2.0) | 2 (4.3) | | 2 (8.0) | 1 (1.9) | |
| | Liver | 15 (30.0) | 8 (17) | | 6 (24.0) | 15 (27.8) | |
| | Lung | 4 (8.0) | 3 (6.4) | | 2 (8.0) | 4 (7.4) | |
| | Lymph nodes | 11 (22.0) | 11 (23.4) | | 3 (12.0) | 18 (33.3) | |

| | | A) females vs males | | | B) marantic first presentation vs known cancer | | |
|---|---|---|---|---|---|---|---|
| | **Level** | **Female** | **Male** | **p** | **Known cancer** | **Marantic first presentation** | **p** |
| | Multiple | 12 (24.0) | 13 (27.7) | | 8 (32.0) | 6 (11.1) | |
| | Others | 7 (14.0) | 5 (10.6) | | 4 (16.0) | 6 (11.1) | |
| **Metastasis (n=141) (%)** | Non- Metastatic | 30(37.5) | 13(21.7) | 0.068 | 14(35.9) | 29(34.9) | 1 |
| | Metastatic | 50(62.5) | 47(78.3) | | 25(64.1) | 54(65.1) | |
| **Marantic presentation before diagnosis of cancer (n=125) (%)** | Known cancer case | 28(38.4) | 14(26.9) | 0.254 | 42 (100.0) | 0 (00.0) | ----- |
| | Marantic as a first presentation | 45(61.6) | 38(73.1) | | 0 (00.0) | 83 (100.0) | |
| **Interval Diagnosis (Months) (mean (SD))** | | 8.25(31.57) | 3.45(12.99) | 0.345 | 20.39(44.18) | 0.32(1.98) | <0.001 |
| **Age (median [IQR])** | | 59.00[49.00,65.00] | 61.00[50.00,67.50] | 0.166 | 57.00[49.25, 66.00] | 61.00[49.50,65.00] | 0.708 |
| **Sex (%)** | F | 81(100.0) | 0 (00.0) | ----- | 28(66.7) | 45(54.9) | 0.285 |
| | M | 0 (00.0) | 63(100.0) | | 14(33.3) | 37(45.1) | |
| **COPD (n=52) (%)** | No | 29(96.7) | 20(90.9) | 0.781 | 10(90.9) | 38(95.0) | 1 |
| | Yes | 1(3.3) | 2(9.1) | | 1(9.1) | 2(5.0) | |
| **Smoke (n=56) (%)** | No | 26(81.2) | 17(70.8) | 0.553 | 9(75.0) | 33(76.7) | 1 |
| | Yes | 6(18.8) | 7(29.2) | | 3(25.0) | 10(23.3) | |
| **Diabetes (n=52) (%)** | No | 25(83.3) | 18(81.8) | 1 | 9(81.8) | 33(82.5) | 1 |
| | Yes | 5(16.7) | 4(18.2) | | 2(18.2) | 7(17.5) | |
| **Obesity (n=53) (%)** | No | 30(96.8) | 22(100.0) | 1 | 11(91.7) | 40(100.0) | 0.519 |
| | Yes | 1(3.2) | 0(0.0) | | 1(8.3) | 0(0.0) | |
| **Dyslipidemia (n=54) (%)** | No | 25(80.6) | 21(91.3) | 0.482 | 9(75.0) | 36(87.8) | 0.528 |
| | Yes | 6(19.4) | 2(8.7) | | 3(25.0) | 5(12.2) | |
| **Hypertension (n=56) (%)** | No | 20(64.5) | 16(64.0) | 1 | 8(61.5) | 27(64.3) | 1 |
| | Yes | 11(35.5) | 9(36.0) | | 5(38.5) | 15(35.7) | |

| | | A) females vs males | | | B) marantic first presentation vs known cancer | | |
|---|---|---|---|---|---|---|---|
| | **Level** | **Female** | **Male** | **p** | **Known cancer** | **Marantic first presentation** | **p** |
| **Embolization event at presentation (n=132) (%)** | No | 6 (7.9) | 9 (16.1) | 0.236 | 8 (19.5) | 6 (7.4) | 0.093 |
| | Yes | 70 (92.1) | 47 (83.9) | | 33 (80.5) | 75 (92.6) | |
| **Embolic event after diagnosis (n=125) (%)** | No | 41 (56.9) | 35 (66) | 0.399 | 27 (65.9) | 46 (56.8) | 0.442 |
| | Yes | 31 (43.1) | 18 (34) | | 14 (34.1) | 35 (43.2) | |
| **Incidental finding of NBTE (%)** | No | 76(93.8) | 56(88.9) | 0.447 | 36(85.7) | 79(95.2) | 0.135 |
| | Yes | 5(6.2) | 7(11.1) | | 6(14.3) | 4(4.8) | |
| **Postmortem diagnosis of NBTE (%)** | No | 55(67.9) | 40(63.5) | 0.706 | 31(73.8) | 63(75.9) | 0.971 |
| | Yes | 26(32.1) | 23(36.5) | | 11(26.2) | 20(24.1) | |
| **Position of vegetation (n=132) (%)** | aortic | 28(37.8) | 20(34.5) | 0.229 | 11(30.6) | 25(32.5) | 0.888 |
| | Left atrium | 0(0.0) | 1(1.7) | | 0(0.0) | 1(1.3) | |
| | mitral | 31(41.9) | 31(53.4) | | 19(52.8) | 37(48.1) | |
| | Multiple | 15(20.3) | 6(10.3) | | 6(16.7) | 14(18.2) | |
| **Valve insufficiency (n=89) (%)** | No | 16(30.2) | 12(33.3) | 0.935 | 9(28.1) | 19(33.3) | 0.787 |
| | yes | 37(69.8) | 24(66.7) | | 23(71.9) | 38(66.7) | |
| **Valve stenosis (n=90) (%)** | No | 48(90.6) | 33(89.2) | 1 | 28(87.5) | 53(91.4) | 0.826 |
| | Yes | 5(9.4) | 4(10.8) | | 4(12.5) | 5(8.6) | |
| **Underwent Intervention (%)** | No | 68(84.0) | 55(87.3) | 0.744 | 36(85.7) | 68(81.9) | 0.778 |
| | Yes | 13(16.0) | 8(12.7) | | 6(14.3) | 15(18.1) | |
| **Type of surgery (%)** | Excision of vegetation | 4(4.9) | 1(1.6) | 0.551 | 3(7.1) | 2(2.4) | 0.203 |
| | None | 68(84.0) | 55(87.3) | | 36(85.7) | 68(81.9) | |
| | replacement | 9(11.1) | 7(11.1) | | 3(7.1) | 13(15.7) | |

| | | A) females vs males | | | B) marantic first presentation vs known cancer | | |
|---|---|---|---|---|---|---|---|
| | **Level** | **Female** | **Male** | **p** | **Known cancer** | **Marantic first presentation** | **p** |
| **Presenting symptoms (n=139) (%)** | Cardiological | 3 (3.9) | 3 (5.5) | 0.552 | 2 (5.1) | 3 (3.6) | 0.582 |
| | Neurological | 60 (77.9) | 36 (65.5) | | 25 (64.1) | 63 (75.9) | |
| | Others | 4 (5.2) | 3 (5.5) | | 3 (7.7) | 4 (4.8) | |
| | Respiratory | 6 (7.8) | 7 (12.7) | | 6 (15.4) | 6 (7.2) | |
| | Vascular | 4 (5.2) | 6 (10.9) | | 3 (7.7) | 7 (8.4) | |
| **Death (144) (%)** | No | 25(31.2) | 21 (33.3) | 1 | 12(29.3) | 32(39.0) | 0.387 |
| | Yes | 55(68.8) | 42 (66.7) | | 29(70.7) | 50(61.0) | |
| **Time to death(days) (median [IQR])** | | 37.94[7.00,122.50] | 33.60[7.00,150.00] | 0.873 | 16.80[3.50,56.00] | 16.80[0.00,45.64] | 0.579 |

**Supplementary Figure S1: PRISMA flowchart of included studies (N=121)/cases (N=144)**

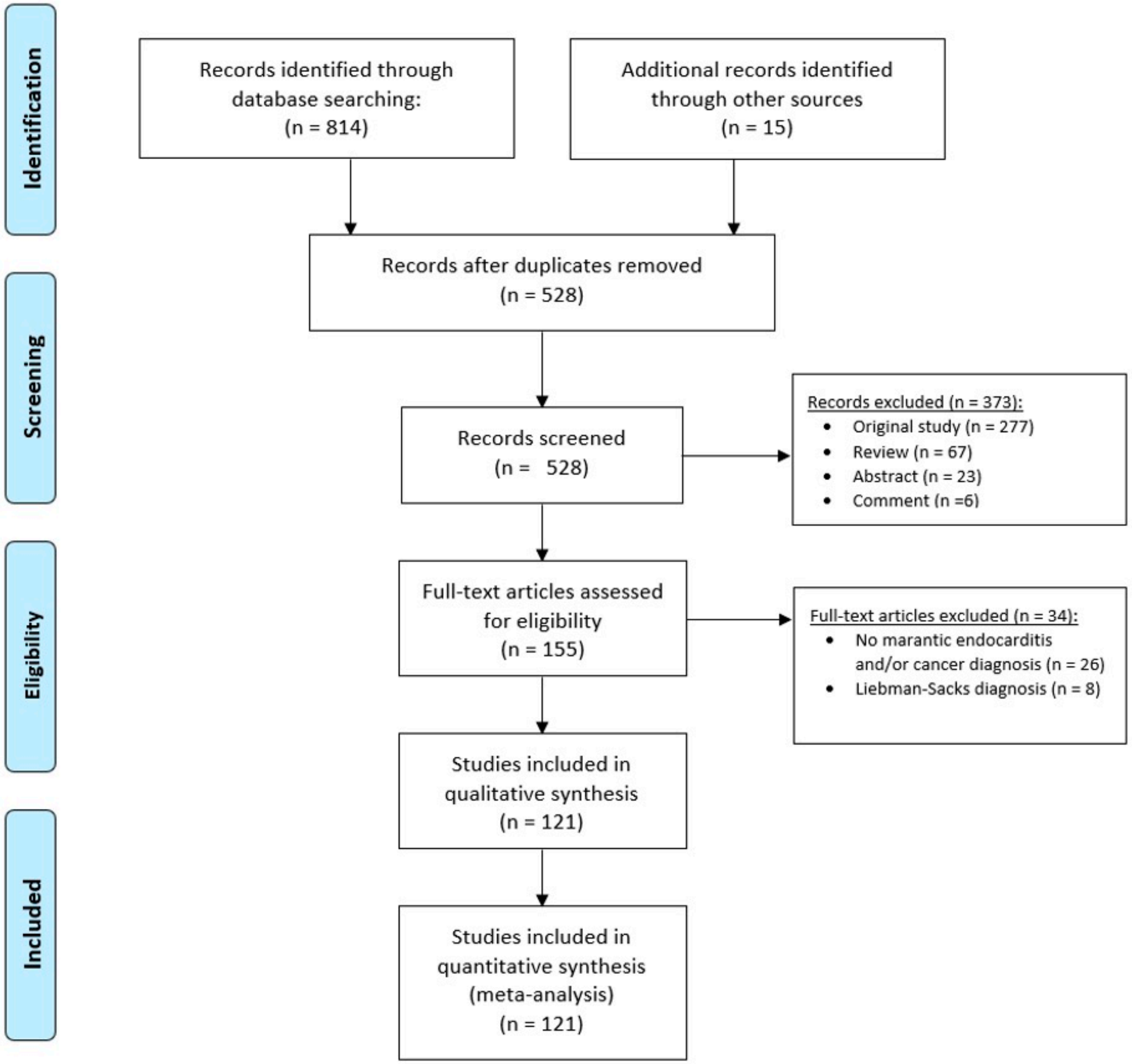

**Supplementary FIGURE S2: Kaplan Meier curves with Estimated survival time among (A) Entire cohort, (B) females vs ales, (C) lung vs others, and (D) marantic 1st presentation vs known cancer, (E) intervention vs no-intervention, (F) metastatic vs non-metastatic cases**

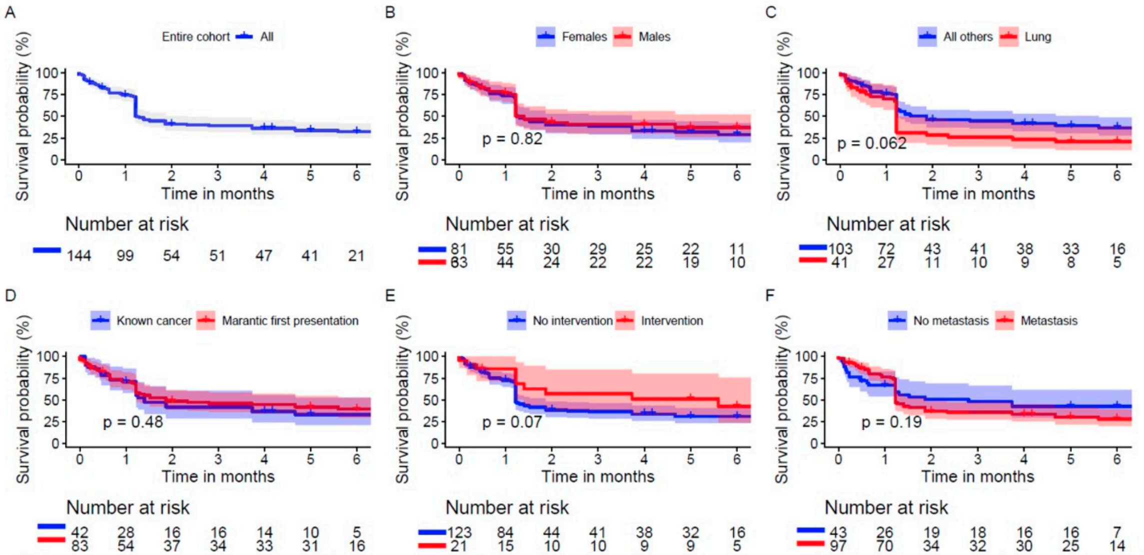


| Variable | Median OS | 1 month (%) | 3 months (%) | 6 months (%) | P value |
|---|---|---|---|---|---|
| • Entire cohort | 1.3[1.2-2.3] | 74.3[67.4-82] | 39.4[31.9-48.6] | 32.3[25-41.7] | ----- |
| ❖ Females | 1.3[1.2-3.7] | 73.4[64.2-83.8] | 38.7[29.1-51.4] | 29.2[20.2-42.4] | 0.82 |
| ❖ Males | 1.4[1.2-6.5] | 76.9[67-88.3] | 41.1[30-56.1] | 37.1[26.4-52.3] | |
| • Other cancers | 1.9[1.2-5.6] | 76[68.1-84.9] | 44.9[36-56.2] | 37.0[28-48.9] | 0.06 |
| • Lung | 1.2[1.2-1.2] | 70.1[57.3-85.9] | 26.0[15.3-44.2] | 20.8[11.2-38.4] | |
| ❖ Known cancer | 1.4[1.2-8.6] | 71[58.4-86.3] | 42.1[29.1-60.8] | 33.5[21.3-52.6] | 0.48 |
| ❖ Marantic first | 1.9[1.2-7.5] | 71.3[62.0-82.0] | 46.1[36.2-58.8] | 39.6[29.7-52.9] | |
| • No intervention | 1.2[1.2-1.9] | 72.6[65.0-81.0] | 36.7[28.9-46.6] | 31.1[23.6-40.9] | 0.07 |
| • Underwent intervention | 5.6[1.4-NA] | 85.7[72.0-100.0] | 57.1[38.4-85.1] | 42.9[24.1-76.1] | |
| ❖ No metastasis | 2.8[1.2-NA] | 66.9[54.1-82.8] | 48.2[34.9-66.6] | 42.8[29.8-61.6] | 0.19 |
| ❖ Metastasis | 1.2[1.2-1.9] | 76.7[68.6-85.7] | 36.1[27.5-47.4] | 28.2[20-39.7] | |

**Supplementary Figure S3.** Number of cases reported per each country

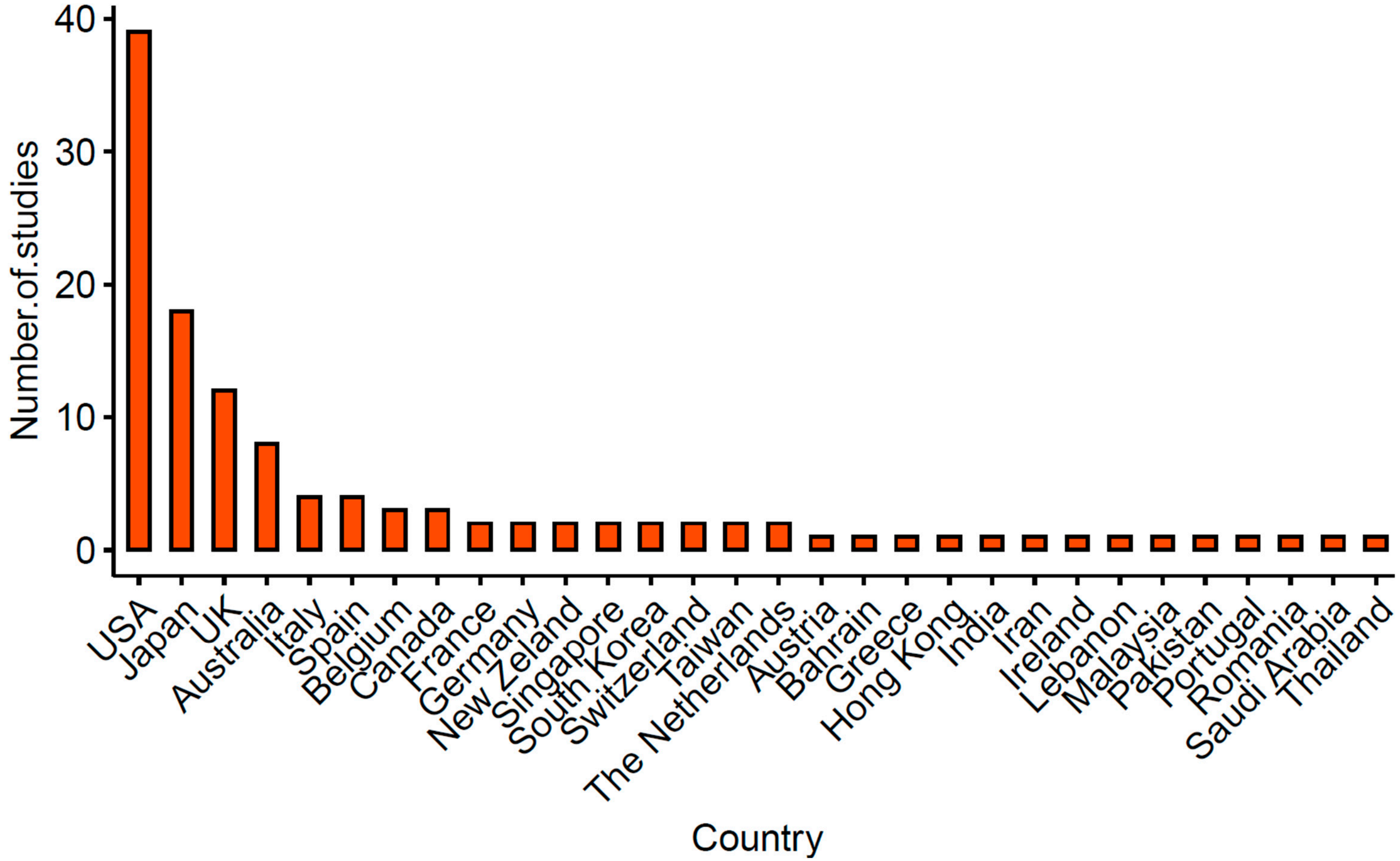

Supplement: Supplementary file 1 [file cancers-15-01848-s001.zip › cancers-2178186-supplementary.pdf]
